# Usefulness of FAPα assessment in bronchoalveolar lavage as a marker of fibrogenesis: results of a preclinical study and first report in patients with idiopathic pulmonary fibrosis

**DOI:** 10.1186/s12931-023-02556-6

**Published:** 2023-10-25

**Authors:** Philomène Lavis, Julien Pingitore, Gilles Doumont, Ani Garabet, Gaetan Van Simaeys, Simon Lacroix, Nicolas Passon, Christophe Van Heymbeek, Coraline De Maeseneire, Justine Allard, Amandine Collin, François Huaux, Christine Decaestecker, Isabelle Salmon, Serge Goldman, Alessandra Kupper Cardozo, Benjamin Bondue

**Affiliations:** 1 Department of Pathology, Hôpital universitaire de Bruxelles (Hôpital Erasme), Université libre de Bruxelles, Brussels, Belgium; 2 I.R.I.B.H.M, Université libre de Bruxelles, Brussels, Belgium; 3 Department of Pneumology, Hôpital universitaire de Bruxelles (Hôpital Erasme), Université libre de Bruxelles, Brussels, Belgium; 4 Center for Microscopy and Molecular Imaging, Université libre de Bruxelles, Brussels, Belgium; 5 Inflammation and Cell Death Signalling group, Experimental Gastroenterology Laboratory and Endotools, Université libre de Bruxelles, Brussels, Belgium; 6 Department of Nuclear Medicine, Hôpital universitaire de Bruxelles (Hôpital Erasme), Université libre de Bruxelles, Brussels, Belgium; 7 Louvain Centre for Toxicology and Applied Pharmacology, Institut de Recherche Expérimentale et Clinique, Université catholique de Louvain, Brussels, Belgium; 8 Laboratory of Image Synthesis and Analysis, Université libre de Bruxelles, Brussels, Belgium; 9 Centre Universitaire inter Régional d’expertise en Anatomie Pathologique Hospitalière, Jumet, Belgium

**Keywords:** Idiopathic pulmonary fibrosis, Fibroblast activation protein, Bronchoalveolar lavage, FAPI, PET scan, Biomarker

## Abstract

**Background:**

Fibroblast activation protein-α (FAPα) is a marker of activated fibroblasts that can be selectively targeted by an inhibitor (FAPI) and visualised by PET/CT imaging. We evaluated whether the measurement of FAPα in bronchoalveolar lavage fluids (BALF) and the uptake of FAPI by PET/CT could be used as biomarkers of fibrogenesis.

**Methods:**

The dynamics of lung uptake of ^18^F-labeled FAPI ([^18^F]FAPI-74) was assessed in the bleomycin mouse model at various time points and using different concentrations of bleomycin by PET/CT. FAPα was measured in BALFs from these bleomycin-treated and control mice. FAPα levels were also assessed in BALFs from controls and patients with idiopathic pulmonary fibrosis (IPF).

**Results:**

Bleomycin-treated mice presented a significantly higher uptake of [^18^F]FAPI-74 during lung fibrinogenesis (days 10 and 16 after instillation) compared to control mice. No significant difference was observed at initial inflammatory phase (3 days) and when fibrosis was already established (28 days). [^18^F]FAPI-74 tracer was unable to show a dose-response to bleomycin treatment. On the other hand, BALF FAPα levels were steeply higher in bleomycin-treated mice at day 10 and a significant dose-response effect was observed. Moreover, FAPα levels were strongly correlated with lung fibrosis as measured by the modified Aschroft histological analysis, hydroxyproline and the percentage of weight loss. Importantly, higher levels of FAPα were observed in IPF patients where the disease was progressing as compared to stable patients and controls. Moreover, patients with FAPα BALF levels higher than 192.5 pg/mL presented a higher risk of progression, transplantation or death compared to patients with lower levels.

**Conclusions:**

Our preclinical data highlight a specific increase of [^18^F]FAPI-74 lung uptake during the fibrotic phase of the bleomycin murine model. The measurement of FAPα in BALF appears to be a promising marker of the fibrotic activity in preclinical models of lung fibrosis and in IPF patients. Further studies are required to confirm the role of FAPα in BALF as biomarker of IPF activity and assess the relationship between FAPα levels in BALF and [^18^F]FAPI-74 uptake on PET/CT in patients with fibrotic lung disease.

**Supplementary Information:**

The online version contains supplementary material available at 10.1186/s12931-023-02556-6.

## Background

Interstitial lung diseases (ILDs) comprise a wide and heterogenous group of pulmonary pathologies with a variable amount of inflammation and/or fibrosis. They can develop as a result of occupational and/or environmental exposure or as manifestation from other diseases (e.g., connective tissue diseases). In a substantial proportion of patients, no etiology is identified and the ILD is categorised as idiopathic [1, 2]. Idiopathic pulmonary fibrosis (IPF) is the most common form of idiopathic interstitial pneumonias, with an estimated incidence of 2.1–6.3 cases/10^5^ person-years [1, 3, 4]. Without treatment, the median survival of IPF patients is 2.5–3.5 years [5]. The diagnosis of IPF is based on a multidisciplinary approach involving clinical, radiological and pathological examinations [4, 6]. After the exclusion of a secondary cause, a high-resolution computed tomography (HRCT) allows to evaluate the lung parenchyma and to eventually identify patterns of usual interstitial pneumonia (UIP) [6]. In the absence of an UIP pattern on HRCT, a lung biopsy is recommended for pathological examination and identification of an UIP pattern [6]. The evolution of the disease is unpredictable. While the majority of patients suffer from a slow degradation of their respiratory function, in some of them, a rapid degradation is observed. Acute exacerbations can also occur and are associated with a poor outcome [5]. A close follow-up of IPF patients is therefore recommended with a monitoring of clinical symptoms, functional parameters such as the forced vital capacity (FVC), the diffusion capacity of the lungs for carbon monoxide (DLCO), six-minute walking tests and thoracic imaging [7]. These elements allow the clinicians to calculate a prognostic score: the GAP score [8, 9]. Various biomarkers were studied to better establish the prognosis of IPF patients. Among them, increased serum concentration of metalloproteinase-7, cancer antigen 19 − 9 and surfactant protein A and D seemed to be associated with a worse prognosis of the disease [10–14]. However, these biomarkers, the GAP score and the HRCT abnormalities mostly reflect the severity of the disease but do not allow the evaluation of the response to antifibrotic treatments nor the intrinsic activity of the fibrotic disease at a selected time point.

A new biomarker candidate of IPF is the fibroblast activation protein-α (FAPα). FAPα is a transmembrane serine protease, expressed at the surface of activated fibroblasts and myofibroblasts [15, 16]. FAPα detection by immunohistochemistry was previously studied in lung tissues and showed that FAPα was not expressed in normal lung tissue nor emphysema but well in fibroblast foci and fibrotic interstitium of IPF patients [17]. Recently, a radiotracer based on a specific enzymatic inhibitor of FAPα (FAPI) was developed and showed a high specificity for cancer-associated fibroblasts both in murine and human tumors [18, 19]. FAPI labelled with Gallium-68 (^68^Ga) was then used to evaluate patients with IPF and lung tumors. Different uptake dynamics were observed in IPF lesions and lung tumors and a positive correlation was observed between lung density and FAPI uptake [20]. These studies indicate that FAPα is a specific marker of lung fibrosis and therefore, the use of FAPα as a biomarker seems to be a promising tool to evaluate fibrogenesis and responses to antifibrotic treatments. To test this hypothesis, we studied FAPα expression in a mouse model of lung fibrosis. More precisely, we evaluated the concentration of FAPα in bronchoalveolar lavage fluids (BALF) and the uptake of FAPI coupled with Fluor-18 ([^18^F]FAPI-74) in bleomycin-treated mice by PET/CT scan. We then evaluated the presence of FAPα in BALFs from IPF patients and correlated the results with the severity of the disease.

## Materials and methods

### Mice and lung fibrosis model

Eight to ten weeks old C57BL/6 female mice (Charles River, Wilmongton, USA) were used for this study. They were kept in a specific-pathogen-free-like environment with access to food and water at will. Lung fibrosis was induced with a single dose of an intratracheal instillation of bleomycin (doses ranging from 0.005U to 0.04U/60μL/mouse) or 60μL of NaCl 0.9% for the controls [21]. Mice were anaesthetised with a mix of Xylazine (0.07 mg/mL, Rompun, Bayer, Germany) and Ketamine (0.36 mg/mL, Nimatek, Dechra, UK). At various time points, mice were sacrificed by exsanguination for lung and BALFs collection. Some mice received orally once a day 60 mg/kg of nintedanib resuspended in 1% Tween 80 or 1% Tween 80 alone, starting one day after the bleomycin instillation for 9 days. All experimental procedures were reviewed and approved by the local Ethics committee for animal welfare of the Université libre de Bruxelles (ULB) (reference: 201,801).

### PET/CT imaging

FAPI was a gift from the society SOFIE/iTheranostics (Dulles, USA). It was coupled with the radioisotope ^18^F, produced by the cyclotron of the department of nuclear medicine of Brussels University Hospital, Brussels, Belgium. From 2.05 to 5.68 MBq of [^18^F]FAPI-74 diluted in 100 to 200μL of NaCl 0.9% were intravenously injected in mice. PET/CT imaging was then performed under isoflurane anaesthesia, from 0 to 110 min after the injection of the radiotracer using a preclinical PET/CT tomograph (NanoScanPET-CT, Mediso, Hungary). The areas of interest (both lungs) were automatically identified on CT then manually corrected for each mouse (Additional Fig. 1A). The muscle uptake was assessed in the right quadriceps. The mean activity in each area of interest was expressed in Bq/mL and was then divided by the ratio A_0_ (injected activity decay-corrected at the start of PET acquisition (Bq) to animal weight (g)) to obtain the mean standardised uptake value (SUVmean). A ratio between the lung uptake and the muscle uptake (ratio lung to muscle or RLM) was also calculated in order to normalise the activity measured in the lung.

### Qualitative and quantitative assessment of fibrosis and fibrogenesis in mice

Lungs from control and bleomycin-treated mice were obtained after dissection. Left lungs were fixed with 4% paraformaldehyde and then embedded in paraffin. Tissues sections (4 μm) were stained with Masson’s trichrome. Ashcroft modified score was used to evaluate fibrotic changes [22]. Eight fields at 20x magnification were analysed. Right lungs were directly stored at -80 °C. Some of these lungs were used to measure hydroxyproline (OH-proline) as previously described [23]. Others were used to perform quantitative reverse transcription polymerase chain reactions (RT-PCR). Briefly, lungs were homogenised at 4 °C and then RNA was extracted and purified with Rneasy Mini Kit (Qiagen, Hilden, Germany). Samples were then reverse transcripted into cDNA and products were analysed by quantitative RT-PCR. The amplification reaction was performed using SYBR green (Bio-Rad, Hercules, USA) and compared with a standard curve. All expression values were corrected with glyceraldehyde-3-phosphate dehydrogenase (GAPDH) as housekeeping gene. Primers are listed in Additional Table 1.

BALFs were obtained at various time points by flushing lungs with 1mL of NaCl 0.9%. Supernatant was collected after a 10-minute centrifugation at 300 g and then stored at -80 °C for further analysis. Measurements of FAPα, monocyte chemoattractant protein-1 (MCP-1) and interleukin 6 (IL-6) on BALF were performed using ELISA kit (R&D systems, Minneapolis, USA). Samples were analysed in duplicates and according to the manufacturer instructions.

### Collection of patients bronchoalveolar lavages and clinical data

BALFs were collected from patients in the process of the diagnosis of their ILD. The IPF diagnosis was thereafter confirmed by a multidisciplinary discussion and based on the 2018 ATS/ERS/JRS/ALAT Clinical Practice Guideline [6]. Control BALF samples were obtained from patients with a pulmonary nodule, from non-infected lung transplant patients with normal HRCT, patient with asthma or chronic cough. Clinical data were gathered from the patient’s medical record including age, gender, body mass index (BMI), tobacco, hypertension, diabetes, and disease progression. The presence of an acute exacerbation was considered as a respiratory deterioration for less than a month associated with new bilateral groundglass opacities or a consolidation on HRCT, not fully explained by cardiac failure or fluid overload [24]. The progression of the disease was based on the 2022 ATS/ERS/JRS/ALAT Clinical Practice Guideline [25]. Patients with a progressive disease presented at least two of the following criteria: worsening of the symptoms, worsening of the fibrotic features on HRCT, more than 5% worsening of the FVC (absolute values) or more than 10% worsening of DLCO in 12 months (absolute values). Plasma and BALF FAPα levels were measured by ELISA (R&D systems, Minneapolis, USA). All samples were analysed in duplicates and according to the manufacturer’s instructions.

### Lung tissue sample collection and immunohistochemistry

Immunohistochemical staining was performed on formalin-fixed paraffin-embedded lung samples from IPF patients. Tissue sections (4 μm) were subjected to standard immunohistochemistry on a PT Link and an Autostainer Link 48 (Agilent Technologies Belgium S.A./N.V., Diegem, Belgium). Briefly, tissue sections were deparaffinized, rehydrated and were subject to antigen retrieval using EnVision FLEX Target Retrieval High pH Solution (EDTA, pH9; Agilent), for 20 min at 97 °C. Then, slides were incubated with peroxydase blocking solution for 5 min and with the rabbit monoclonal anti-FAPα antibody for 30 min (dilution 1:200, Abcam, Cambridge, UK). The slides were washed and incubated with the EnVision + System-HRAP labelled Polymer anti-rabbit Ig antibody for 30 min (Agilent). Immunostaining was revealed by incubation with diaminobenzidine and hydrogen peroxydase (Agilent). The slides were counterstained with hematoxylin for 5 min, dehydrated and mounted. They were digitized at 20x using a NanoZoomer 2.0 HT (Hamamatsu, Hamamatsu-City, Japan) before assessment.

### Statistical analysis

All data were tested for normality using the Shapiro-Wilk test and according to the distribution, parametric or non-parametric tests were applied. Differences between the groups were assessed by one-way ANOVA or Kruskal-Wallis test. Holm’s Sidak or Dunn’s tests were used as post-hoc tests. When only two groups were compared, student t or Mann-Whitney tests were performed. Two-way ANOVA was used for the comparison of 2 independent variables followed by Bonferroni’s correction for multiple comparisons. Data are reported as mean ± standard deviation (SD) or median with interquartile range. For correlation analysis, parametric Pearson or non-parametric Spearman correlation were performed and data are reported as Pearson or Spearman r and p-value. Kaplan-Meier survival curves were generated and a log-rank test was applied to compare the two groups of patients. The analysis was done on Graph Pad Prism 8 (San Diego, USA) and all significance levels were fixed at 0.05.

## Results

### The pulmonary uptake of [^18^F]FAPI-74 is a marker of fibrogenesis in bleomycin-treated mice

**Fig. 1:**
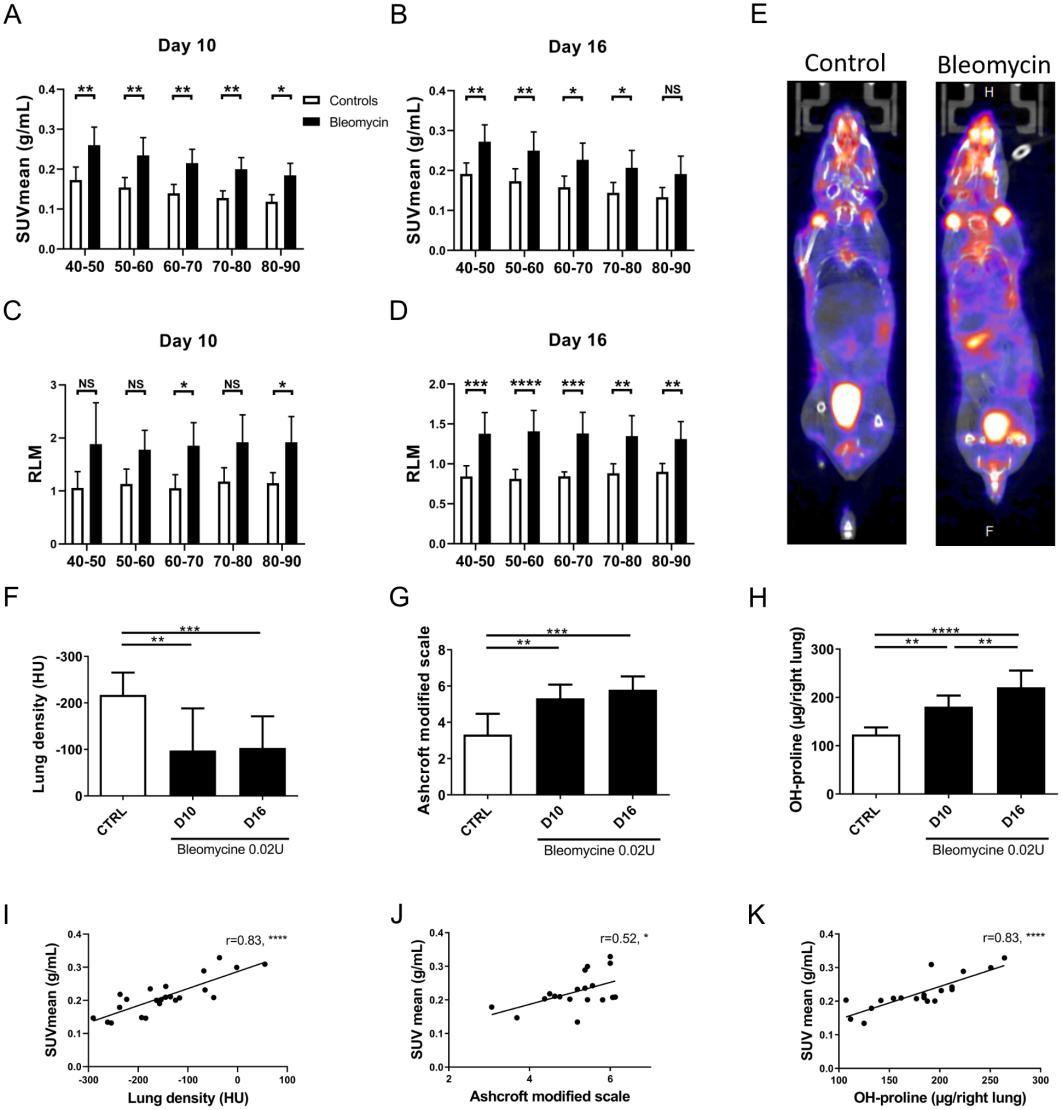
Bleomycin-treated mice present a higher uptake of [^18^F]FAPI-74 compared to control mice: Assessment of lung [^18^F]FAPI-74 uptake in control (white column bars) and bleomycin-treated (black column bars) mice at days 10 and 16 after instillation. Acquisitions were performed between 40 and 90 min after the radiotracer injection with subanalyses every 10 min. The lung uptake was respectively evaluated by the calculation of the mean standardized uptake value (SUVmean) (**A-B**) and the ratio between the uptake in lungs and the uptake in muscle (RLM) (**C-D**). Data are presented as mean with standard deviation and statistics analysis was performed using Two-Way ANOVA followed by Bonferroni correction test for multiple comparisons. **E.** Representative uptake of [^18^F]FAPI-74 at day 10 after instillation in a control (left) and a bleomycin-treated mice (right). **F-H.** Evaluation of the development of pulmonary fibrosis in bleomycin-treated mice (black column bars) compared to control mice (white column bars) at day 10 and 16 after instillation by the measurement of lung density (**F**), the Ashcroft modified scale (**G**) and the hydroxyproline (OH-proline) content of the right lung (**H**). Data are presented as mean with standard deviation and statistics analysis was performed using One-Way ANOVA followed by Holm-Sidak’s post-hoc test for multiple comparisons. **I-K.** Fibrosis parameters were correlated with the SUVmean obtained between 50 and 60 min after the radiotracer injection. The r corresponds to the Pearson coefficient for parametric correlation. Controls: n = 4–5; bleomycin-treated: n = 7–10. *: p < 0.05; **: p < 0.01; ***: p < 0.001; ****: p < 0.0001

To determine the optimal window for the [^18^F]FAPI-74 PET acquisition, we analysed the dynamics of the tracer uptake in control mice and mice treated with bleomycin (0.01 or 0.02U/mouse) at day 10 post-instillation. Continuous (dynamic) acquisitions from time zero (injection of the radiotracer) up to 90 min (with sub-analyses of 10 min) were performed. An early and intense uptake of the tracer in the lungs and the heart was observed in all mice during the first 10 min, probably linked to the vascular distribution of [^18^F]FAPI-74. Afterwards, retention of the radiotracer was observed in the lungs of bleomycin-treated mice. The optimal window for analysis of [^18^F]FAPI-74 uptake was between 40 and 90 min post-injection as tracer uptake remained stable in the lungs (Additional Fig. 1B). We observed a higher and significant uptake of [^18^F]FAPI-74 in bleomycin-treated mice (0.02U/mouse) compared to control mice at 10 and 16 days post-instillation (Fig. 1A-B; Table 1).

**Table 1:** Evolution of the lung uptake of [^18^F]FAPI-74 at days 10 and 16 after instillation

	Day 10			Day 16		
	Controls (n = 4)	Bleomycin (n = 7)	p-value	Controls (n = 5)	Bleomycin (n = 7)	p-value
SUVmean 40–50 (g/mL)	0.17 ± 0.03	0.26 ± 0.04	**0.0029**	0.19 ± 0.03	0.27 ± 0.04	**0.0037**
SUVmean 50–60 (g/mL)	0.15 ± 0.02	0.23 ± 0.04	**0.0019**	0.17 ± 0.03	0.25 ± 0.05	**0.0065**
SUVmean 60–70 (g/mL)	0.14 ± 0.02	0.21 ± 0.03	**0.0037**	0.16 ± 0.03	0.23 ± 0.04	**0.0182**
SUVmean 70–80 (g/mL)	0.13 ± 0.02	0.20 ± 0.03	**0.0064**	0.14 ± 0.03	0.21 ± 0.04	**0.0379**
SUVmean 80–90 (g/mL)	0.12 ± 0.02	0.18 ± 0.03	**0.0142**	0.13 ± 0.02	0.19 ± 0.04	0.0650

The standardised uptake value (SUVmean) was measured between 40 and 90 min after injection with subanalyses of 10 min. Data are presented as mean ± Standard deviation. Statistical analysis was performed using a Two-Way ANOVA followed by Bonferroni correction test for multiple comparisons. Bold p-value indicate a significant difference

This difference was observed during all time frames at day 10. At day 16, the significant time windows were 40–50, 50–60, 60–70 and 70–80 min post injection of the [^18^F]FAPI-74. As bleomycin-treated mice tend to lose weight, [^18^F]FAPI-74 lung uptake was normalised by the uptake in the quadriceps (RLM, Fig. 1C-D) to correct for weight-loss. The RLM confirmed a significant and higher uptake of [^18^F]FAPI-74 at day 10 during the time periods of 60–70 and 80–90 min after injection and for all time windows analysed at day 16 (Fig. 1C and D).

Bleomycin-treated mice developed a significant fibrosis, as confirmed by the evaluation of the mean lung density on CT scan (controls: -217.2 ± 47.9 HU; bleomycin-treated mice at day 10: -97.6 HU ± 90.5; bleomycin-treated mice at day 16: -103.0 HU ± 68.0, mean ± SD, p < 0.01, Fig. 1F), the Ashcroft modified scale (controls: 3.32 ± 1.15; bleomycin-treated mice at day 10: 5.32 ± 0.76; bleomycin-treated mice at day 16: 5.80 ± 0.73, mean ± SD, p < 0.001, Fig. 1G) and the measurement of lung OH-proline content (controls: 123.5 μg ± 14.3; bleomycin-treated mice at day 10: 180.9 μg ± 22.9; bleomycin-treated mice at day 16: 220.9 μg ± 34.8, mean ± SD, p < 0.0001, Fig. 1H). Moreover, a significant and positive correlation was observed between the SUVmean measured between 50 and 60 min after the radiotracer injection and the lung density (r = 0.83, p < 0.0001, Fig. 1I), the Ashcroft modified scale (r = 0.52, p < 0.05, Fig. 1J) and lung content of OH-proline (r = 0.83, p < 0.0001, Fig. 1K).

**Fig. 2:**
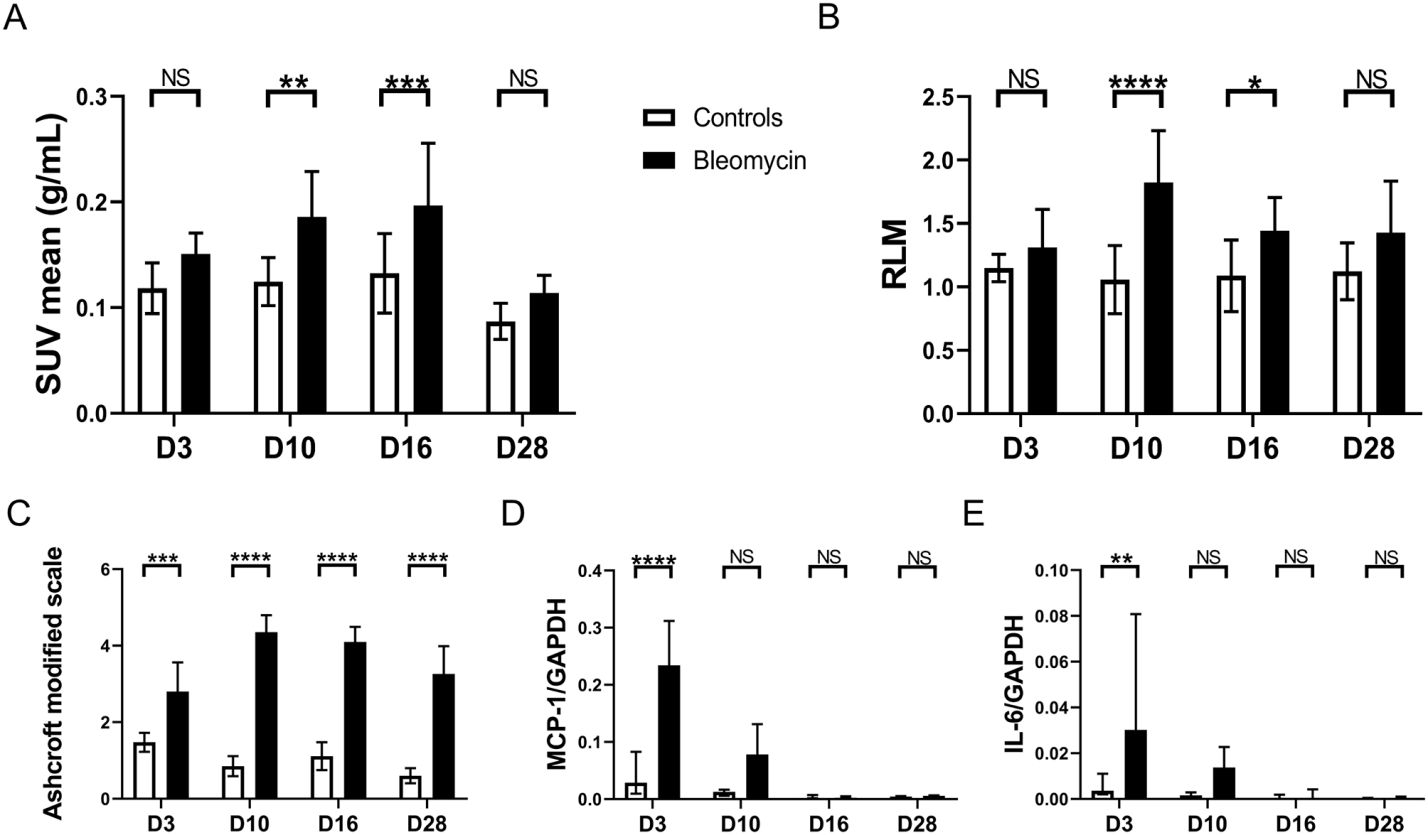
The pulmonary uptake of [^18^F]FAPI-74 is a marker of fibrogenesis in bleomycin-treated mice: **A-B.** Time-course of the [^18^F]FAPI-74 lung uptake measured between 60 and 70 min after the radiotracer injection at day 3 (D3), day 10 (D10), day 16 (D16) and day 28 (D28) after instillation in bleomycin-treated (black column bars) and control mice (white column bars). **C.** Evaluation of lung fibrosis at D3, D10, D16 and D28 using the Ashcroft modified scale. Data are presented as mean with standard deviation and statistical analysis was performed using Two-Way ANOVA followed by Bonferroni’s correction test for multiple comparisons. **D-E.** Evaluation of the inflammatory response based on the RNA expression of MCP-1 and IL-6, normalized by GAPDH. Data are presented as median with interquartile range and statistical analysis was performed using Two-Way ANOVA followed by Bonferroni correction test for multiple comparisons. Controls n = 4–10; bleomycin-treated: n = 6–15. *: p < 0.05; **: p < 0.01; ***: p < 0.001; ****: p < 0.0001

To determine the time-course of lung [^18^F]FAPI-74 uptake, PET/CT imaging were performed at days 3, 10, 16 and 28 post-instillation in controls and bleomycin-treated mice (0.02U/mouse). The acquisitions were done between 50 and 90 min with 10 min subanalysis post-injection of the radiotracer. A significantly higher uptake of the radiotracer, evaluated by the SUVmean and the RLM, was observed at days 10 and 16 with no statistical difference at days 3 and 28 (Fig. 2A-B; Table 2).

**Table 2:** Kinetic of lung uptake of [^18^F]FAPI-74 in control compared to bleomycin-treated mice

	SUVmean controls 60–70 (g/mL)	SUVmean bleomycin 60–70 (g/mL)	p-value
Day 3	0.12 ± 0.02	0.15 ± 0.02	0.2974
Day 10	0.11 ± 0.01	0.16 ± 0.03	**0.0405**
Day 16	0.11 ± 0.03	0.15 ± 0.06	**0.0207**
Day 28	0.09 ± 0.02	0.11 ± 0.02	0.4464

Data show the standardised uptake value (SUVmean) measured between 60 and 70 min after the tracer injection and are presented as mean ± Standard deviation. Statistical analysis was performed using a Two-Way ANOVA followed by Bonferroni correction test for multiple comparisons. Bold p-value indicate a significant difference

The development of a significant fibrosis was confirmed by the Ashcroft modified scale (day 3: controls 1.47 ± 0.25 versus bleomycin 2.80 ± 0.76, p < 0.001; day 10: controls 0.85 ± 0.26 versus bleomycin 4.35 ± 0.44, p < 0.0001; day 16: controls 1.11 ± 0.36 versus bleomycin 4.10 ± 0.39, p < 0.0001; day 28: controls 0.60 ± 0.19 versus bleomycin 3.3 ± 0.73, p < 0.0001, mean ± SD, Fig. 2C). The development of an early inflammatory response in the bleomycin model was confirmed by a significant increase of MCP-1 and IL-6 mRNA levels at day 3 in bleomycin-treated mice compared to controls (p < 0.01 and p < 0.0001, respectively). No significant difference was observed at days 10, 16 and 28 for these pro-inflammatory genes (Fig. 2D-E).

We have then performed a dose response experiment (0.005U, 0.01U and 0.02U/mouse) at day 10 post-instillation of bleomycin and analysed lung [^18^F]FAPI-74 uptake. The SUVmean did not significantly differ between the 4 groups (controls: 0.22 g/mL ± 0.02; bleomycin 0.005U: 0.21 g/mL ± 0.04; bleomycin 0.01U: 0.22 g/mL ± 0.05; bleomycin 0.02U: 0.24 g/mL ± 0.04, mean ± SD, p = NS, Additional Fig. 2). Regarding the RLM, a significant increase of [^18^F]FAPI-74 lung uptake was observed for the highest bleomycin dose (0.02U) compared to control mice (controls: 1.4 ± 0.16 versus bleomycin 0.02U: 2.02 ± 0.41, mean ± SD, p < 0.05) but no significant difference was observed with the lower doses (bleomycin 0.005U: 1.73 ± 0.28 and bleomycin 0.01U: 1.49 ± 0.23, mean ± SD, Additional Fig. 2B). The measurement of OH-proline showed a trend toward higher values in mice treated with higher bleomycin doses. However, it was only significant between the dose of 0.005 and 0.02 (controls: 117.20 μg ± 4.88; bleomycin 0.005U: 112.2 μg ± 13.27; bleomycin 0.01U: 135.1 μg ± 26.73; bleomycin 0.02U: 152.3 μg ± 24.98, mean ± SD, p < 0.05, Supplemental Fig. 2C). A moderate correlation was seen between the lung content of OH-proline and the SUVmean and the RLM (r = 0.49, p < 0.05 and r = 0.53, p < 0.05, respectively). On the contrary, we observed a dose response effect for the Ashcroft modified scale (controls: 1.08 ± 0.26; bleomycin 0.005U: 3.8 ± 0.18; bleomycin 0.01U: 4.35 ± 0.46; bleomycin 0.02U: 5.26 ± 0.49, mean ± SD, p < 0.0001, Supplemental Fig. 2D). No correlation was highlighted between the SUVmean and the Ashcroft modified scale (r = 0.18, p = NS) but a moderate correlation was observed between the RLM and the Ashcroft modified scale (r = 0.55, p < 0.05).

### The measurement of FAPα in BALF is a marker of fibrogenesis in bleomycin-treated mice

**Fig. 3:**
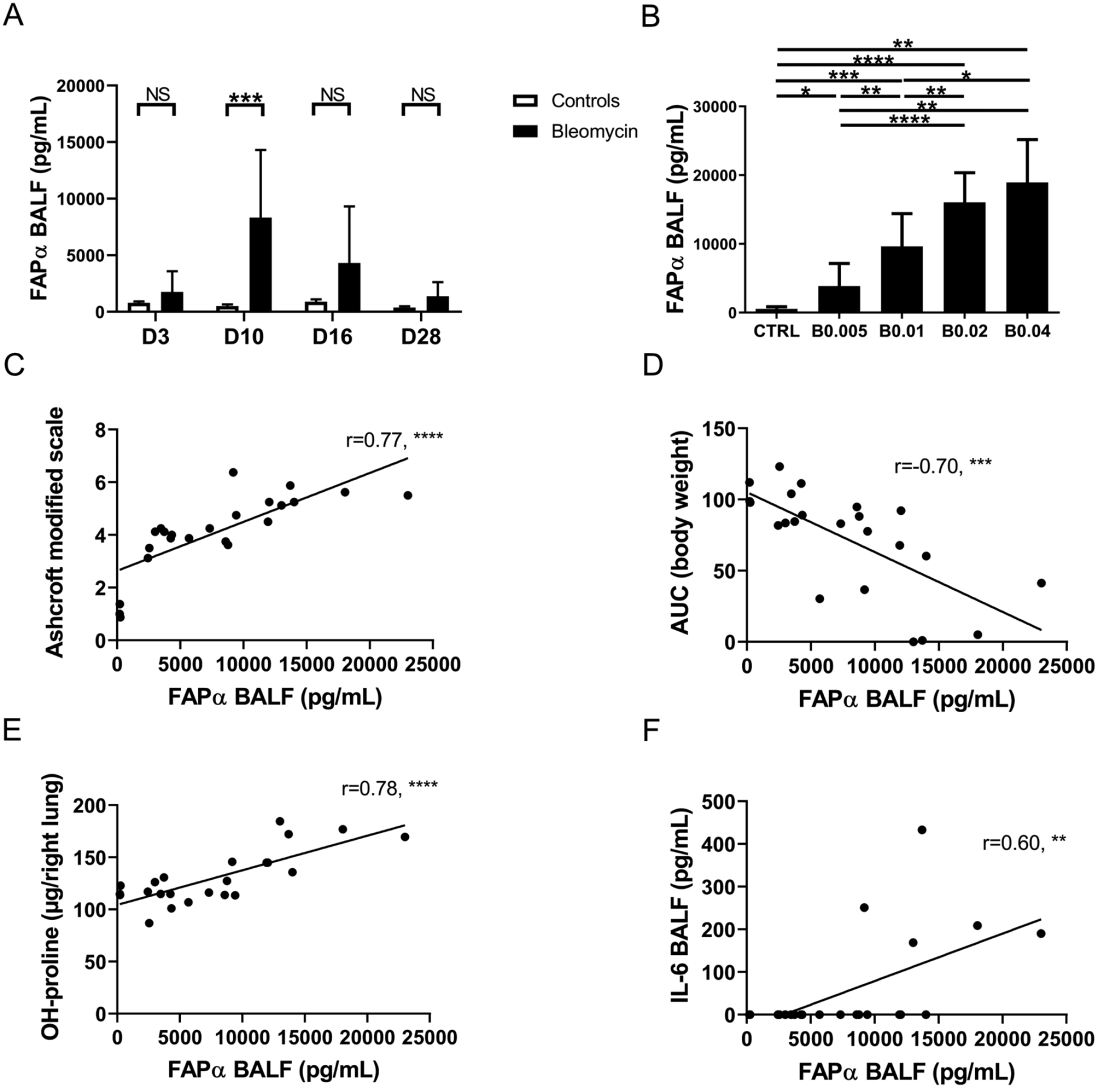
The measurement of FAPα in BALF is a marker of lung fibrogenesis: (**A**) Time-course of the concentration of FAPα in BALF day 3 (D3), day 10 (D10), day 16 (D16) and day 28 (D28) after instillation in bleomycin (black column bars) or 0,9% NaCl (control) mice (white column bars). Data are presented as mean with standard deviation and statistical analysis was performed using Two-Way ANOVA followed by Bonferroni correction test for multiple comparisons. Controls: n = 5–6; bleomcyin-treated mice: n = 6–8. (**B**) Effect of various doses of bleomycin (0,005, 0,01, 0,02 and 0,04U/mouse) on the FAPα BALF concentration at day 10 after bleomycin instillation. Data are presented as mean with standard deviation and statistical analysis was performed using One-Way ANOVA followed by Holm-Sidak’s post-hoc test. Controls: n = 24; 0.005U bleomycin-treated mice (B0.005), n = 13; 0.01U bleomycin-treated mice (B0.01), n = 13; 0.02U bleomycin-treated mice (B0.02), n = 14; 0.04U bleomycin-treated mice (B0.04), n = 7. **C-F.** Correlations between FAPα BALF concentration, the Ashcroft modified scale (**C**), the percentage of weight loss (expressed by the area under the curve (AUC)) (**D**), the concentration of hydroxyproline (OH-proline) (**E**) and the concentration of interleukin-6 (IL6) (**F**). The r corresponds to the Pearson coefficient for parametric correlation. *: p < 0.05; **: p < 0.01; ***: p < 0.001; ****: p < 0.0001

In order to evaluate whether FAPα in BALF could be used as a biomarker of fibrogenesis, we measured it in BALF obtained from the mice experiments described above. Interestingly, FAPα was significantly increased in BALF from bleomycin-treated mice (0.02U/mouse) at day 10 post-instillation compared to control mice (controls: 528.0 pg/mL (277.0-639.0) versus bleomycin: 11455.0 pg/mL (275.0-13831.0), median (CI 95%), p < 0.001). No significant difference was observed at days 3, 16 and 28 post-instillation with a trend for higher FAPα in BALF at day 16 (Fig. 3A).

**Fig. 4:**
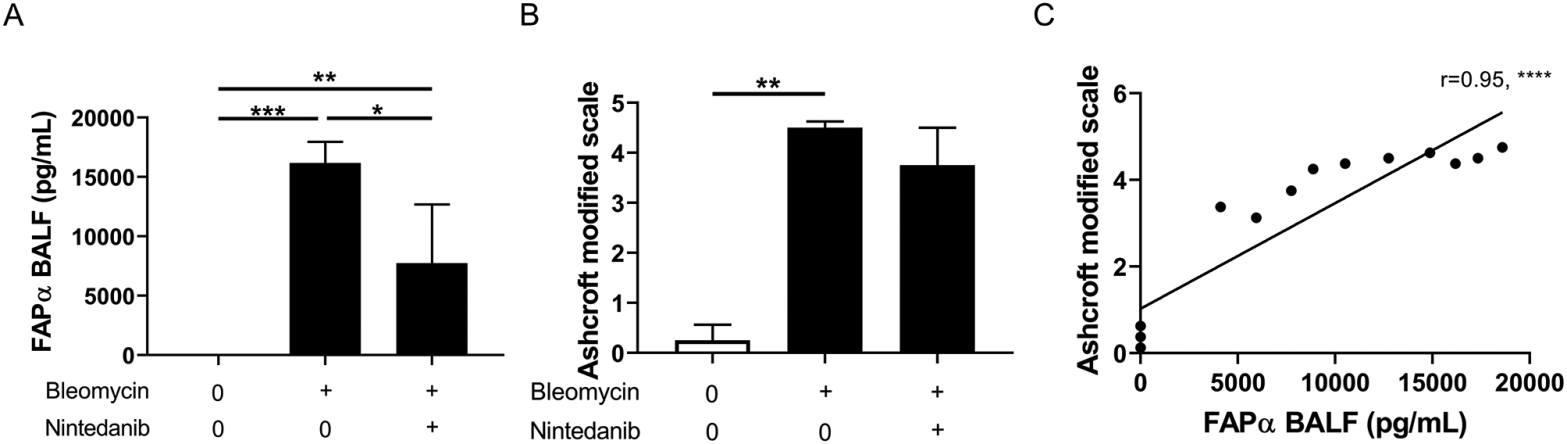
Effect of an antifibrotic treatment on FAPα levels in BALFs from bleomycin-treated mice: (**A**) Measurement of the concentration of FAPα in BALFs from control mice (CTRL) and bleomycin-treated mice (0.01U/mouse) eight days after instillation. Bleomycin-treated mice received nintedanib (NTD) or the vehicle orally once a day. Data are presented as mean with standard deviation and statistical analysis was performed using One-Way ANOVA followed by Holm-Sidak’s post-hoc test. (**B**) Evaluation of the Ashcroft modified scale. (**C**) Correlation of Ashcroft modified scale and FAPα concentration in BALF (**C**). Data are presented as median with interquartile range and statistic analysis was performed using Kruskal-Wallis test followed by Dunn’s post-hoc test. The r represents the Spearman r for non-parametric correlation. CTRL (0,9%NaCl) : n = 4; nintedanib: n = 5; vehicle (1% Tween 80): n = 5. *: p < 0.05; **: p < 0.01; ***: p < 0.001; ****: p < 0.0001

Using a dose range of bleomycin (0.005U, 0.01U, 0.02U and 0.04U/mouse), a dose-dependent increase of FAPα in BALF was observed at day 10 post-instillation (Fig. 3B). Furthermore, a strong correlation between FAPα concentration in BALF and the Ashcroft modified scale (r = 0.77, p < 0.0001), the concentration of OH-proline in the right lung (r = 0.78, p < 0.0001) and the percentage of weight loss (r=-0.70, p < 0.001) was observed. Interestingly, the correlation with the concentration of IL-6 in BALF was only moderate (r = 0.60, p < 0.001) (Fig. 3C-F). A moderate correlation between the RLM and FAPα in BALF was seen (r = 0.56, p < 0.05). Moreover, FAPα was measured in BALF from mice instilled with bleomycin (0.01U/mouse) and then treated for 1 week with nintedanib or vehicle. BALF from mice instilled with NaCl 0.9% and receiving vehicle for 1 week were chosen as controls. FAPα was undetectable in control mice and was significantly decreased in mice receiving nintedanib compared to the non-treated mice (controls: 0 pg/mL ± 0; nintedanib-treated: 8641.0 pg/mL ± 4209.0; vehicle-treated: 14,746 pg/mL ± 3938, mean ± SD, p < 0.001, Fig. 4A). The Ashcroft modified scale confirmed that bleomycin-treated mice developed a significant fibrosis compared to controls. Although a trend for a lower score was observed in nintedanib-treated as compared to the vehicle-treated mice, no significant difference was observed (controls: 0.31 ± 0.24; nintedanib-treated: 3.85 ± 0.64; vehicle-treated: 4.47 ± 0.18, mean ± SD, p < 0.01, Fig. 4B). Interestingly, a strong correlation was observed between the FAPα concentration in BALF and the Ashcroft modified scale (r = 0.95, p < 0.0001, Fig. 4C).

### FAPα is a marker of fibrosis and progression in IPF patients

BALFs were collected from 27 patients with IPF as part of the current protocol for disease diagnosis and from 2 patients during an acute exacerbation according to the 2016 International Working Group [24]. As controls, we selected 19 patients where bronchoalveolar lavages were performed for the assessment of a pulmonary nodule, during bronchoscopy for chronic cough, or performed as routine screening for graft rejection after lung transplantation. Baseline characteristics are summarised in Additional Table 2. IPF patients were older than controls (68.5 ± 9.5 years versus 56.2 ± 12.5, mean ± SD, p < 0.001) and more controls presented hypertension (13 controls (68.4%) versus 9 IPF patients (31.0%)). No difference in clinical characteristic was observed regarding gender, BMI, diabetes, tobacco consumption and lung function tests between IPF patients and controls. Regarding the severity of IPF patients, the mean predicted FVC was assessed at 83.46% ± 18.00, the median predicted DLCO at 51.50% (40.00–58.00) and the GAP score at 3.46 ± 1.36.

**Fig. 5:**
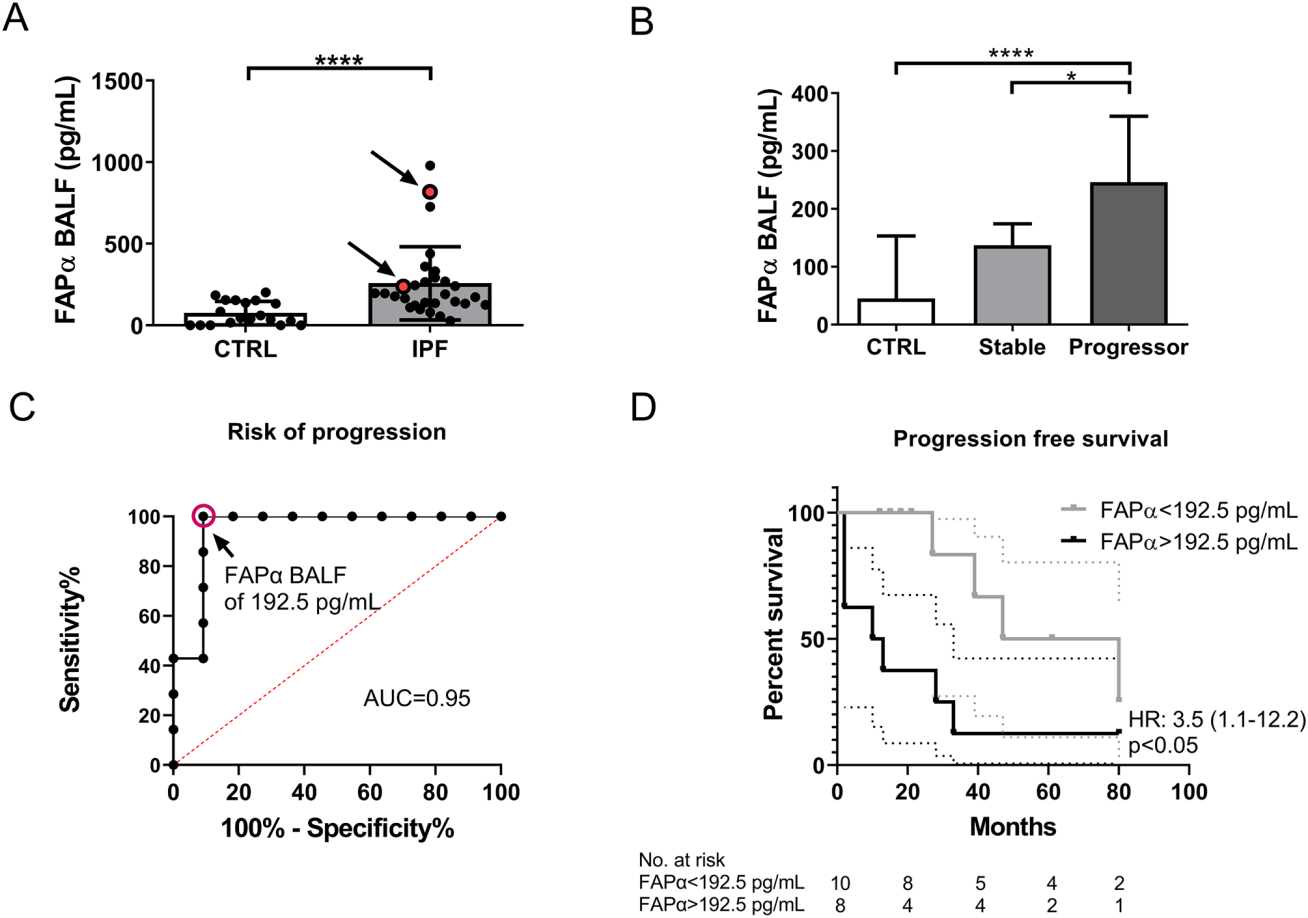
FAPα concentration in BALF is higher in IPF patients: (**A**) Measurement of FAPα in BALF from patients with idiopathic pulmonary fibrosis (IPF) compared to controls (CTRL). Red points correspond to patients with an acute exacerbation. Data are presented as median with interquartile range and statistical analysis was performed using Mann-Whitney test. CTRL: n = 19 and IPF patients: n = 29. (**B**) Comparison of the FAPα BAL concentration between stable and IPF patients with a progressive phenotype (progressor) and controls (CTRL). Data are presented as median with interquartile range and statistical analysis was performed using Kruskal-Wallis test followed by Dunn’s post-hoc test. CTRL: n = 19; stable IPF patients n = 10; progressor IPF patients n = 7. (**C**) ROC analysis of the risk of progression based on FAPα BALF concentration. (**D**) Kaplan-Meier curves showing the progression free survival of IPF patients with lower or higher FAPα BALF concentration of 192.5 pg/mL. Progression was defined as having either disease progression as defined in the 2022 guidelines, a lung transplantation or death. *: p < 0.05; ****: p < 0.0001

FAPα was significantly higher in IPF patients compared to controls (190.0 pg/mL (135.0-265.0) versus 45.0pg/mL (0.0-153.0), median (95% CI), p < 0.0001) (Fig. 5A).

Seventeen IPF patients had a follow-up of at least 12 months and could be categorised as stable or progressors according to the 2022 ATS/ERS/JRS/ALAT Clinical Practice Guideline [25]. Patients with a progressive disease presented at least two of the following criteria: worsening of the symptoms, worsening of the fibrotic features on HRCT, more than 5% worsening of the FVC (absolute values) or more than 10% worsening of DLCO in 12 months (absolute values). Ten patients (58.8%) were considered as stable and seven as progressors (41.2%). Progressors patients presented higher and significant levels of BALF FAPα compared to controls and stable patients (progressors: 246.0 pg/mL (195.0-439.0) versus controls: 45.0 pg/mL (0.0-153.0) and stable: 137.0 pg/mL (122.0-178.0), median (95% CI), p < 0.0001 and p < 0.05 respectively). No significant difference was observed between controls and stable patients (Fig. 5B). In the two patients with an acute exacerbation, high FAPα concentrations were measured (819.0 pg/mL and 237.0 pg/mL, respectively, Fig. 5A, red points).

A receiver operating characteristic (ROC) curve was generated to evaluate the predictive value of FAPα BALF concentration on the risk of progression. It showed that FAPα levels higher than 192.5 pg/mL predicted the risk of progression with a sensitivity of 100.0%, a specificity of 90.9%, a positive predictive value of 87.5% and a negative predictive value of 100.0% (Fig. 5C).

The progression free survival was further analysed in IPF patients with a follow-up of at least 12 months and separated according to the FAPα BALF cut-off concentration of 192.5 pg/mL. The endpoint was considered as the time before a progression according to the 2022 guidelines [25], a lung transplantation or death and regrouped as the term survival. Patients with the highest FAPα levels presented a significantly lower survival compared to patients with FAPα levels lower than 192.5 pg/mL (hazard ratio: 3.50 (0.10-12.25), p < 0.05, Fig. 5D).

### FAPα is expressed by activated fibroblasts, myofibroblasts and hyperplastic alveolar cells

**Fig. 6:**
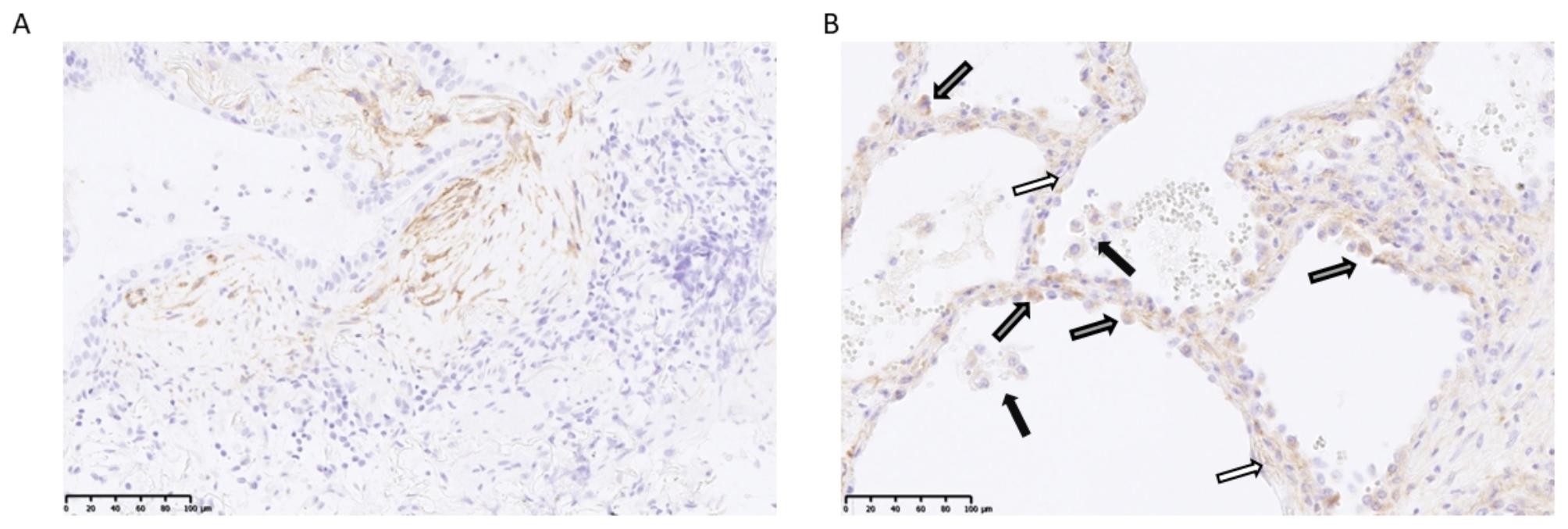
Representative anti-FAPα staining in a fibroblast foci (**A**), fibrotic interstitium (white arrows) and hyperplastic alveolar cells (gray arrows) (**B**). Absence of alveolar macrophages staining (black arrows). Field magnification: 200x

As previously demonstrated, a staining for FAPα was observed in activated fibroblasts and myofibroblasts in fibrotic interstitium and fibroblast foci (Fig. 6A) [17]. Moreover, a staining was also highlighted in hyperplastic alveolar cells that was not previously described (Fig. 6B). No staining in alveolar macrophages was observed (Fig. 6B).

## Discussion

The present study aimed to evaluate the role of FAPα assessment either by PET/CT using a ^18^F-labelled FAPα inhibitor and by dosing FAPα BALF levels by ELISA. Regarding [^18^F]FAPI-74 imaging in an in vivo mouse model of lung fibrosis, we showed that this radiotracer is a useful tool to investigate fibrotic activity. Indeed, a higher and significant uptake of [^18^F]FAPI-74 is observed at days 10 and 16 post-bleomycin instillation, the period when fibrogenesis occurs in the lung, but neither at day 3, during the early inflammatory phase nor at day 28, during the late fibrotic stage of the model when fibrosis is installed but without further progression. Our results are consistent with recent reports in the literature. Rosenkrans et al. evaluated the lung uptake of [^68^Ga]FAPI-46 in a mouse bleomycin model and observed a higher and significant lung uptake of the radiotracer at 7 and 14 days with the highest uptake seen at day 14 [26]. Another study successfully used [^68^Ga]FAPI-46 to discriminate lesions of lung carcinoma and lung fibrosis in ILD patients [20]. Finally, Yang et al. used [^68^Ga]FAPI-04 and observed a higher uptake of the radiotracer in lung from IPF patients compared to controls and the FAPI SUVtotal was negatively correlated with DLCO [27]. Therefore, [^18^F]FAPI-74 PET imaging could provide additional information to respiratory function and HRCT, since these methods evaluate the impact and severity of the disease rather than the intrinsic fibrotic activity. Thus, [^18^F]FAPI-74 PET imaging could be an important tool to determine the activity of the disease, its prognosis and to support the decision to prescribe a specific anti-fibrotic therapy, especially in non IPF fibrotic disease (for example ILD secondary to inflammatory disease) helping to decide between the initiation of an antiinflammatory drug alone or in combination with an antifibrotic one.

[^18^F]FAPI-74 PET/CT imaging appears to be a better biomarker of the fibrosis activity as compared to [^18^F]FDG. Indeed our group previously showed, in the bleomycin model, that [^18^F]FDG PET/CT imaging had a significant and higher uptake in lungs from bleomycin-treated mice during the fibrotic phase of the model but also when fibrotic activity is not yet present or already stable, i.e. during the inflammatory and the late fibrotic phases [23]. Here we show that [^18^F]FAPI-74 PET imaging is reflecting the current active fibrogenesis as its uptake is only observed during the fibrogenesis period. Another advantage compared to [^18^F]FDG is that [^18^F]FAPI-74 PET imaging is unaffected by changes in lung density. Indeed, even if [^18^F]FDG uptake in IPF patients is associated with decrease in respiratory function and clinical severity [28, 29], when a correction for lung density is applied, no significant correlation between [^18^F]FDG uptake and one-year prognosis was observed in a cohort of IPF patients [30]. [^18^F]FDG PET/CT was also unable to assess the early response to antifibrotic treatment [31]. On the contrary, [^18^F]FAPI-74 PET imaging appears more specific to fibrogenesis and is not affected by changes in lung density as no uptake is noticed in dense organs such as the brain or the liver in normal condition.

Our study identified one important limitation of [^18^F]FAPI-74 PET/CT imaging in the bleomycin model. The determination of regions of interest in mice is challenging due to the presence of non-specific uptake by the thoracic wall and spine (Additional Fig. 1C). The difficulty to discriminate lung uptake from the uptake in the surrounding tissue reduced the precision of measurements. Of note, as the human lung is bigger, this issue is not applicable in patients with fibrotic ILDs. This is probably one of the reasons why we failed to demonstrate a clear dose-response effect on the [^18^F]FAPI-74 lung uptake in our mouse model and the fact that only at the highest dose of bleomycin (0.02U) a higher uptake of the tracer was measurable in the lungs.

Beside PET/CT evaluation of [^18^F]FAPI-74 lung uptake, we identified in the BALF of bleomycin-treated mice a significant increase of the FAPα protein as assessed by ELISA. In line with our results, Yang et al. reported an increase of the protein expression of FAPα in homogenised lungs from bleomycin-treated mice as compared to controls [27]. Interestingly, contrary to what we observed for the pulmonary uptake of [^18^F]FAPI-74, a strong and significant dose response effect was observed on FAPα levels in BALF in experiments with variable doses of bleomycin. Furthermore, strong correlations between FAPα concentration in BALF and the Ashcroft modified scale, the concentration of OH-proline in the lungs, and the weight loss were identified. This suggest that BALF concentration of FAPα has a better sensitivity than [^18^F]FAPI-74 PET/CT imaging, in the bleomycin model. The lung fibrosis is commonly evaluated by the measurement of OH-proline. However, no dose-response effect was observed in our experiment at day 10 after bleomycin instillation, on the contrary of FAPα BALF concentration. We thus think that FAPα is a better and early marker of fibrosis. BALF FAPα concentration appears also better than the Ashcroft modified scale. Indeed, this latter is a semi-quantitative method to evaluate fibrosis with an inter-observer variability [22]. Moreover, no significant difference was observed in the Ashcroft modified scale between mice receiving or not nintedanib as antifibrotic treatment, on the contrary of a significant decrease of their BALF FAPα concentration. Altogether, BALF FAPα concentration appears then useful to monitor therapeutic response in preclinical models of lung fibrosis and probably more sensible than the measurement of OH-proline and the Ashcroft modified scale.

In humans, we further confirmed the interest of FAPα measurement in BALF. To our knowledge, we are the first to report that the concentration of FAPα in BALF is significantly higher in IPF patients. Moreover, the higher levels were found in patients with a progressive form of IPF and, in two cases, during an acute exacerbation. Accordingly, our data showed an interesting prognostic role of FAPα measurement in BALF using a cut-off value of 192.5 pg/mL. This confirms the potential of this biomarker in the follow-up of patients affected by a disease characterised by its unpredictable course made of periods of stability, steady progression and acute worsening. The measurement of FAPα in BALF by ELISA seems a useful, easy and cheap way to evaluate the fibrotic activity in the lungs. It could be implemented in the management of ILD patients and have prognostic value at the diagnosis. The immunohistochemistry analysis allowed us to identify the cell populations expressing FAPα, including activated fibroblasts and myofibroblasts, as already described [17] but also alveolar epithelial cells that appeared hyperplastic. This population could be responsible of the higher FAPα concentration in BALF, following desquamation in the alveolar lumen or through direct secretion. Given that there is a soluble form of FAPα, also known as α2-antiplasmin cleaving enzyme [32], the increased expression of FAPα on alveolar epithelial cells or activated fibroblasts and myofibroblasts could lead to the higher FAPα concentration in BALF. On the contrary, alveolar macrophages, the main cell population found in BALF [6] did not seem to express FAPα. Arnold et al. identified a population of cancer-associated macrophages expressing FAPα and that presented characteristics of alternatively activated macrophages [33]. However, to our knowledge, FAPα is not expressed by alveolar macrophages and based in our data they does not seem to be the source of the FAPα detected in BALF. Further studies should be performed to identify the cell population(s) that lead to higher FAPα BALF levels.

Our study has some limitations. IPF patients and controls were not matched by age and IPF patients were significantly older than controls. However, the impact of age on FAPα levels is probably limited as no strong association has been demonstrated in the limited available literature: in a cohort of patients with oesophageal squamous cell carcinoma, no significant difference of plasma FAPα concentration was observed between patients older or younger than 62 years old [34]. Also, another group measured FAPα concentration in plasma samples from patients with ST-elevation myocardial infarction (STEMI) and healthy donors. Contrary to our results, the group with the higher FAPα levels in plasma (healthy donors) was younger than STEMI patients [35]. Another important limitation is that our study was performed on a very small cohort of patients from a single center. Therefore, multicenter studies should be initiated to validate the use of BALF FAPα as a biomarker of lung fibrosis and prognosis of IPF. Unfortunately, bronchoalveolar lavage is an invasive act associated with some risk of exacerbation of the underlying disease [36, 37]. Therefore, it could not be repeated periodically to assess changes in the fibrotic activity of the disease. Further research could focus on the determination of FAPα in other biological fluids such as plasma, induced sputum or even exhaled breath condensates.

## Conclusions

Our preclinical data demonstrate the specific increase of [^18^F]FAPI-74 lung uptake during the fibrotic phase of the bleomycin model. These data announce a promising clinical translation into a valuable PET/CT evaluation of patients with fibrotic ILD. We also identify that determination of FAPα concentration in BALF could be an outstanding marker of the fibrotic activity in preclinical lung fibrosis models and in IPF patients. Further studies are required to confirm these initial results.

### Electronic supplementary material

Below is the link to the electronic supplementary material.

Supplementary Material 1

## Data Availability

The dataset used and/or analysed during this study are available from the corresponding author on reasonable request.

## List of abbreviations

^18^F: Fluor-18
^68^Ga: Gallium-68
BALF: Bronchoalveolar lavage fluid
BMI: Body mass index
DLCO: Diffusion capacity of the lungs for carbon monoxide
FAPα: Fibroblast activation protein-α
FAPI: Inhibitor of FAPα
FVC: Forced vital capacity
GAPDH: Glyceraldehyde-3-phosphate dehydrogenase
HRCT: High-resolution computed tomography
ILD: Interstitial lung disease
IL-6: Interleukin-6
IPF: Idiopathic pulmonary fibrosis
KL-6: Krebs von den Lungen-6
MCP-1: Monocyte chemoattractant protein-1
OH-proline: Hydroxyproline
RLM: Ratio lung to muscle
RT-PCR: Quantitative reverse transcription polymerase chain reaction
SD: Standard deviation
STEMI: ST-elevation myocardial infarction
SUVmean: Standardised uptake value
UIP: Usual interstitial pneumonia

## References

[1] Hilberg O, Hoffmann-Vold A-M, Smith V, Bouros D, Kilpeläinen M, Guiot J (2022). Epidemiology of interstitial lung diseases and their progressive-fibrosing behaviour in six european countries. ERJ Open Res.

[2] Wijsenbeek M, Suzuki A, Maher TM (2022). Interstitial lung diseases. Lancet.

[3] Demedts M, Wells AU, Antó JM, Costabel U, Hubbard R, Cullinan P (2001). Interstitial lung diseases: an epidemiological overview. Eur Respir J Suppl.

[4] Travis WD, Costabel U, Hansell DM, King TE, Lynch DA, Nicholson AG (2013). An official American thoracic Society/European respiratory society statement: update of the international multidisciplinary classification of the idiopathic interstitial pneumonias. Am J Respir Crit Care Med.

[5] Ley B, Collard HR, King TE (2011). Clinical course and prediction of survival in idiopathic pulmonary fibrosis. Am J Respir Crit Care Med.

[6] Raghu G, Remy-Jardin M, Myers JL, Richeldi L, Ryerson CJ, Lederer DJ (2018). Diagnosis of idiopathic pulmonary fibrosis. An Official ATS/ERS/JRS/ALAT Clinical Practice Guideline. Am J Respir Crit Care Med.

[7] Fernández Fabrellas E, Peris Sánchez R, Sabater Abad C, Juan Samper G (2018). Prognosis and Follow-Up of idiopathic pulmonary fibrosis. Med Sci (Basel).

[8] Ley B, Ryerson CJ, Vittinghoff E, Ryu JH, Tomassetti S, Lee JS (2012). A multidimensional index and staging system for idiopathic pulmonary fibrosis. Ann Intern Med.

[9] Ley B, Bradford WZ, Weycker D, Vittinghoff E, du Bois RM, Collard HR (2015). Unified baseline and longitudinal mortality prediction in idiopathic pulmonary fibrosis. Eur Respir J.

[10] Bauer Y, White ES, de Bernard S, Cornelisse P, Leconte I, Morganti A (2017). MMP-7 is a predictive biomarker of disease progression in patients with idiopathic pulmonary fibrosis. ERJ Open Res.

[11] Barlo NP, van Moorsel CHM, Ruven HJT, Zanen P, van den Bosch JMM, Grutters JC (2009). Surfactant protein-D predicts survival in patients with idiopathic pulmonary fibrosis. Sarcoidosis Vasc Diffuse Lung Dis.

[12] Kinder BW, Brown KK, McCormack FX, Ix JH, Kervitsky A, Schwarz MI (2009). Serum surfactant protein-A is a strong predictor of early mortality in idiopathic pulmonary fibrosis. Chest.

[13] Maher TM, Oballa E, Simpson JK, Porte J, Habgood A, Fahy WA (2017). An epithelial biomarker signature for idiopathic pulmonary fibrosis: an analysis from the multicentre PROFILE cohort study. Lancet Respir Med.

[14] Karampitsakos T, Juan-Guardela BM, Tzouvelekis A, Herazo-Maya JD (2023). Precision medicine advances in idiopathic pulmonary fibrosis. EBioMedicine.

[15] Fitzgerald AA, Weiner LM (2020). The role of fibroblast activation protein in health and malignancy. Cancer Metastasis Rev.

[16] Juillerat-Jeanneret L, Tafelmeyer P, Golshayan D (2021). Regulation of fibroblast activation Protein-α expression: focus on intracellular protein interactions. J Med Chem.

[17] Acharya PS, Zukas A, Chandan V, Katzenstein A-LA, Puré E (2006). Fibroblast activation protein: a serine protease expressed at the remodeling interface in idiopathic pulmonary fibrosis. Hum Pathol.

[18] Loktev A, Lindner T, Mier W, Debus J, Altmann A, Jäger D (2018). A tumor-imaging Method Targeting Cancer-Associated fibroblasts. J Nucl Med.

[19] Loktev A, Lindner T, Burger E-M, Altmann A, Giesel F, Kratochwil C (2019). Development of fibroblast activation protein-targeted Radiotracers with Improved Tumor Retention. J Nucl Med.

[20] Röhrich M, Leitz D, Glatting FM, Wefers AK, Weinheimer O, Flechsig P (2022). Fibroblast activation protein-specific PET/CT imaging in Fibrotic interstitial lung Diseases and Lung Cancer: a translational exploratory study. J Nucl Med.

[21] Moore BB, Hogaboam CM (2008). Murine models of pulmonary fibrosis. Am J Physiol Lung Cell Mol Physiol.

[22] Hübner R-H, Gitter W, El Mokhtari NE, Mathiak M, Both M, Bolte H (2008). Standardized quantification of pulmonary fibrosis in histological samples. Biotechniques.

[23] Bondue B, Sherer F, Van Simaeys G, Doumont G, Egrise D, Yakoub Y (2015). PET/CT with 18F-FDG- and 18F-FBEM-labeled leukocytes for metabolic activity and leukocyte recruitment monitoring in a mouse model of pulmonary fibrosis. J Nucl Med.

[24] Collard HR, Ryerson CJ, Corte TJ, Jenkins G, Kondoh Y, Lederer DJ (2016). Acute Exacerbation of Idiopathic Pulmonary Fibrosis. An International Working Group Report. Am J Respir Crit Care Med.

[25] Raghu G, Remy-Jardin M, Richeldi L, Thomson CC, Inoue Y, Johkoh T (2022). Idiopathic pulmonary fibrosis (an update) and progressive pulmonary fibrosis in adults: an Official ATS/ERS/JRS/ALAT Clinical Practice Guideline. Am J Respir Crit Care Med.

[26] Rosenkrans ZT, Massey CF, Bernau K, Ferreira CA, Jeffery JJ, Schulte JJ (2022). [68 Ga]Ga-FAPI-46 PET for non-invasive detection of pulmonary fibrosis disease activity. Eur J Nucl Med Mol Imaging.

[27] Yang P, Luo Q, Wang X, Fang Q, Fu Z, Li J et al. Comprehensive Analysis of Fibroblast activation protein expression in interstitial Lung Diseases. Am J Respir Crit Care Med n d;207:160–72. 10.1164/rccm.202110-2414OC 10.1164/rccm.202110-2414OC PMC9893314 35984444

[28] Justet A, Laurent-Bellue A, Thabut G, Dieudonné A, Debray M-P, Borie R (2017). [18F]FDG PET/CT predicts progression-free survival in patients with idiopathic pulmonary fibrosis. Respir Res.

[29] Lee EYP, Wong CS, Fung SL, Yan PK, Ho JCM (2014). SUV as an adjunct in evaluating disease activity in idiopathic pulmonary fibrosis - a pilot study. Nucl Med Commun.

[30] Castiaux A, Van Simaeys G, Goldman S, Bondue B (2018). Assessment of 18F-FDG uptake in idiopathic pulmonary fibrosis: influence of lung density changes. Eur J Hybrid Imaging.

[31] Bondue B, Castiaux A, Van Simaeys G, Mathey C, Sherer F, Egrise D (2019). Absence of early metabolic response assessed by 18F-FDG PET/CT after initiation of antifibrotic drugs in IPF patients. Respir Res.

[32] Lee KN, Jackson KW, Christiansen VJ, Lee CS, Chun J-G, McKee PA (2006). Antiplasmin-cleaving enzyme is a soluble form of fibroblast activation protein. Blood.

[33] Arnold JN, Magiera L, Kraman M, Fearon DT (2014). Tumoral Immune suppression by Macrophages expressing fibroblast activation protein-alpha and heme Oxygenase-1. Cancer Immunol Res.

[34] Liao Y, Xing S, Xu B, Liu W, Zhang G (2017). Evaluation of the circulating level of fibroblast activation protein α for diagnosis of esophageal squamous cell carcinoma. Oncotarget.

[35] Tillmanns J, Fraccarollo D, Galuppo P, Wollert KC, Bauersachs J (2017). Changes in concentrations of circulating fibroblast activation protein alpha are associated with myocardial damage in patients with acute ST-elevation MI. Int J Cardiol.

[36] Sakamoto K, Taniguchi H, Kondoh Y, Wakai K, Kimura T, Kataoka K (2012). Acute exacerbation of IPF following diagnostic bronchoalveolar lavage procedures. Respir Med.

[37] Hiwatari N, Shimura S, Takishima T, Shirato K (1994). Bronchoalveolar Lavage as a possible cause of Acute Exacerbation in Idiopathic Pulmonary Fibrosis Patients. Tohoku J Exp Med.

