# Supplementary material for: Usefulness of FAPα assessment in bronchoalveolar lavage as a marker of fibrogenesis: results of a preclinical study and first report in patients with idiopathic pulmonary fibrosis
Source: Respir Res. 2023 Oct 25;24:254. doi: 10.1186/s12931-023-02556-6 (PMC10601150; doi:10.1186/s12931-023-02556-6)
Supplement: Supplementary file 1 — Supplementary Material 1 [file 12931_2023_2556_MOESM1_ESM.docx]

**
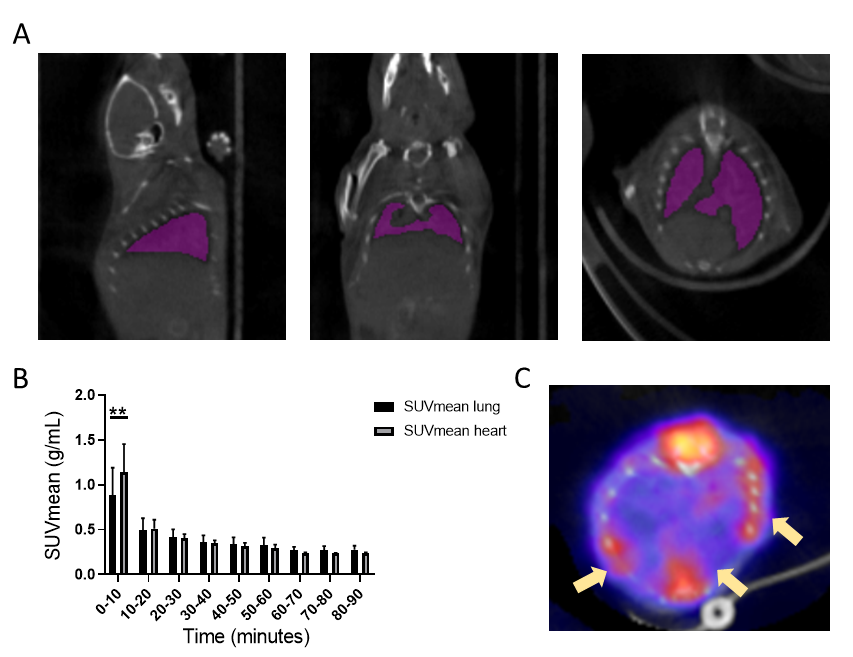
**

**Additional Figure 1.** *Illustration of the selection of regions of interest in both lungs and the cinetic of* [^18^F]FAPI-74 *thoracic uptake.* **A.** Selection of the region of interest in the three representative sections (sagittal, coronal, transversal). **B.** There is a vascular distribution of [^18^F]FAPI-74 during the first 10 minutes after injection and then the uptake in the lungs is stable between 40 and 90 minutes. N=6. Data are presented as mean with standard deviation and statistical analysis were performed by two-way ANOVA followed by Bonferroni’s post-hoc test. **: p<0.01. **C.** Illustration of the uptake of [^18^F]FAPI-74 in thoracic wall and spine (yellow arrows), leading to difficulties to select regions of interest and inaccuracies in the SUVmean measurements.


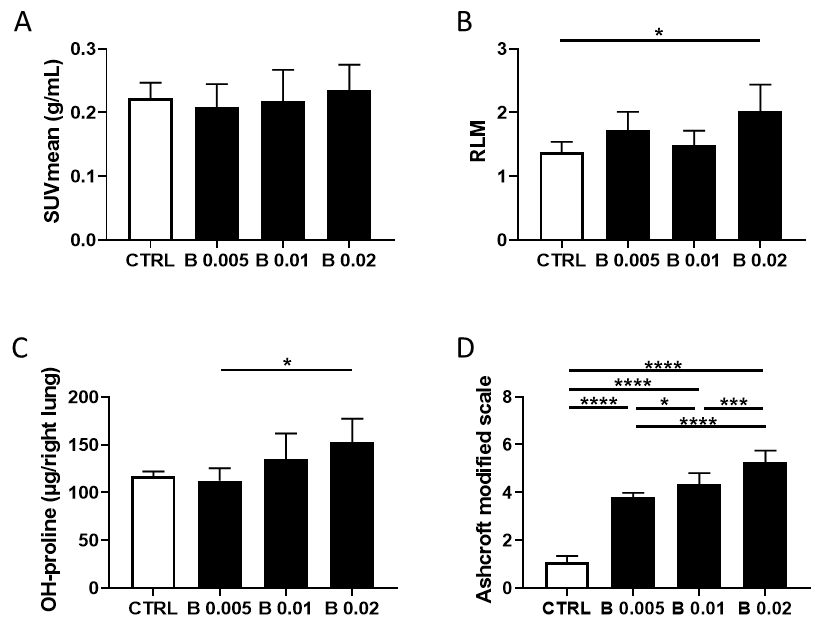


**Additional Figure 2.** *Absence of dose-response effect on the uptake of* [^18^F]FAPI-74*:* **A-B.** Lung [^18^F]FAPI-74 uptake in the dose response model (bleomycin 0,005, 0,01 and 0,02U/mouse) expressed as the SUVmean (A) and lung-to-muscle ratio (RLM) (B) at day 10 after bleomycin instillation. Controls (CTRL): n=3; 0.005U bleomycin-treated mice (B0.005), n=5; 0.01U bleomycin-treated mice (B0.01), n=5; 0.02U bleomycin-treated mice (B0.02), n=5. **C-D.** Right lung content of hydroxyproline measurement (C) and evaluation of the Ashcroft modified scale (D) in the dose-response model. CTRL: n=3; B0.005: n=7; B0.01: n=7; B0.02: n=6. Data are presented as mean with standard deviation and statistical analysis was performed using One-Way ANOVA followed by Holm-Sidak’s post-hoc test. *: p<0.05; ***: p<0.001; ****: p<0.0001.

**Additional Table 1. Sequence of primers used for quantitative RT-PCR**

| **Genes** |  | **Primer sequences** |
| --- | --- | --- |
| GAPDH | Forward | 5’- GCCTGGAGAAACCTGCCAAGTATGA-3’ |
|  | Reverse | 5’- AACCTGGTCCTCAGTGTAGCCC-5’ |
| IL6 | Forward | 5’-GCTACCAAACTGGATATAATCAGGA-3’ |
|  | Reverse | 5’-CCAGGTAGCTATGGTACTCCAGAA-3’ |
| MCP-1 | Forward | 5’-GTGTTGGCTCAGCCAGATGC-3’ |
|  | Reverse | 5’-GACACCTGCTGCTGGTGATCC-3’ |

GAPDH: Glyceraldehyde-3-phosphate dehydrogenase; IL6: Interleukin 6; MCP-1: Monocyte chemoattractant protein-1.

**Additional Table 2. Baseline characteristics.**

|  | **Controls (n=19)** | **IPF patients (n=29)** | **p-value** |
| --- | --- | --- | --- |
| **Age (years)** | 56.2 ± 12.5 | 68.5 ± 9.5 | **0.0003** |
| **Gender (M/F)** | 11/8 | 23/6 | 0.1931 |
| **Hypertension n (%)** | 13 (68.4) | 9 (31.0) | **0.0177** |
| **Diabetes n (%)** | 1 (5.3) | 4 (13.8) | 0.6351 |
| **BMI (kg/m²)** | 26.1 ± 4.9 | 26.8 ± 4.4 | 0.5924 |
| **Tobacco (no/yes/former)** | 3/3/12 | 4/3/20 | 0.8365 |
| **FVC (L)** | 2.90 ± 0.89 | 2.95 ± 0.94 | 0.8695 |
| **Predicted FVC (%)** | 84.11 ± 25.30 | 83.46 ± 18.00 | 0.9211 |
| **DLCO (ml/min/mmHg)** | 16.78 (7.49-20.21) | 11.34 (9.46-14.12) | 0.2213 |
| **Predicted DLCO (%)** | 60.00 (33.00-81.00) | 51.50 (40.00-58.00) | 0.1862 |

Data are presented as mean ± Standard deviation or median (Confidence interval 95%). BMI: body mass index. Statistical analysis was performed using t-test for non-categorical variables. Fisher’s exact test was applied for categorical variables or Chi-square test if more than 2 categorical variables. Bold p-value indicate a significant difference.
